# Partial Internal Biliary Diversion: A Solution for Intractable Pruritus in Progressive Familial Intrahepatic Cholestasis Type 1

**DOI:** 10.4103/1319-3767.80387

**Published:** 2011

**Authors:** Ramaswamy Ganesh, Natarajan Suresh, Malathi Sathiyasekeran, Priya Ramachandran

**Affiliations:** Kanchi Kamakoti CHILDS Trust Hospital & The CHILDS Trust Medical Research Foundation, Nungambakkam, Chennai, India

**Keywords:** Biliary diversion, cholestasis, low GGT

## Abstract

Biliary diversion offers a potential option for intractable pruritus in children with chronic cholestatic disorders. Progressive familial intrahepatic cholestasis (PFIC) is an inherited disorder of impaired bile acid transport and excretion, which presents with jaundice and pruritus in the first few months of life and progresses to cirrhosis by infancy or adolescence. We report a child with PFIC type 1 who underwent internal biliary diversion for intractable pruritus and was relieved of his symptoms.

Progressive familial intrahepatic cholestasis (PFIC) type 1 is a subtype of a group of inherited progressive cholestatic disorders and is characterized by intrahepatic cholestasis, intense pruritus, normal or low gamma glutamyl transpeptidase (GGT), and characteristic ȌByler bile” on electron microscopy (EM). Liver transplantation is recommended for PFIC; however, this may not be the solution in PFIC 1 where apart from the liver, there is also involvement of the intestine and the pancreas. Biliary diversion offers significant relief in intractable pruritus not responding to conventional medications.[1,2] We report a child with PFIC type 1 with disturbing pruritus who underwent internal biliary diversion.

## CASE REPORT

**Figure 1:**
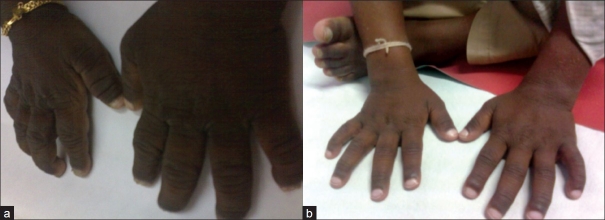
(a) Hands showing broad stubby fingers with grossly thickened, rough, and lichenified skin; (b) normal hands and fingers following biliary diversion

**Figure 2:**
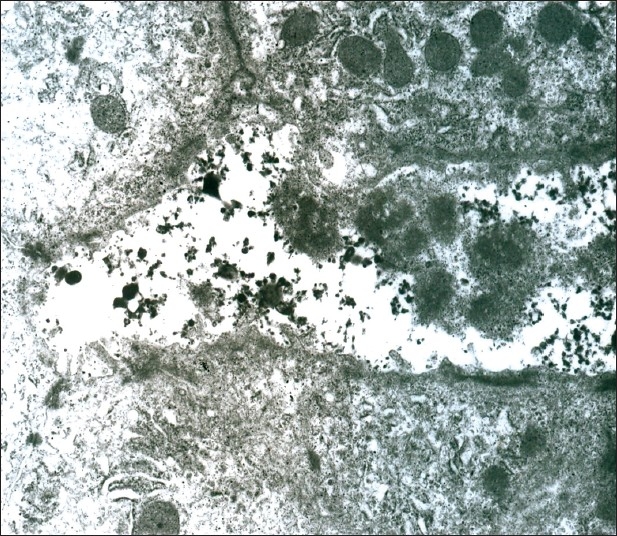
Electron microscopy of liver showing distended bile canaliculi with coarse and granular bile

**Figure 3:**
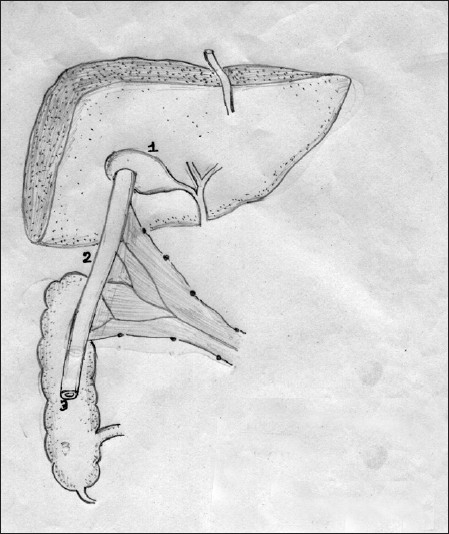
Technique of cholecystojejunocolic anastomosis (1) Gall bladder; (2) Loop of jejunum; (3) Mid ascending colon

A 7-year-old male child presented with persistent jaundice, high colored urine, intractable pruritus, and growth retardation since the age of 6 months. He was the first born of third degree consanguinity with a birth weight of 3.5 kg. Jaundice was persistent from infancy; however, itching was the most distressing symptom and over the years, it had become intractable, requiring a cocktail of medications. There was no history of ascites, gastrointestinal bleeds, irritability, fractures, night blindness, or lethargy. He was hospitalized once for epistaxis and treated for coagulopathy. On examination, he was apathetic, stunted (height 90 cm, <5^th^centile), and undernourished (weight 13 kg, <5^th^centile) with a Body mass index of 16. He had no dysmorphic features but was icteric with scratch marks on his face and ears. His hands and feet were enlarged without rachitic changes or xanthomata. The fingers and toes were broad and stubby with hyperpigmented, thick, rough, and lichenified skin [Figure 1a]. Liver and spleen were firm and enlarged. Cardiovascular and respiratory system examinations were normal. His complete blood count was normal. Total bilirubin was 16.8 mg/dl; direct, 12.8 mg/dl; serum alkaline phosphatase, 774 IU/l (100-644); GGT, 20 IU/l (0-26); alanine transaminase, 169 IU/l (0-45); aspartate transaminase, 61 IU/l (0-45); serum cholesterol was 100 mg/dl (70-122); alpha fetoprotein, 1.56 ng/ml; and Serum bile acids was 120 *μ*mol/L (0-10). Total protein and serum albumin were normal. Ultrasound showed hepatomegaly with normal echo texture. Hepatobiliary scan revealed decreased uptake and delayed excretion. Liver biopsy showed bland cholestasis and on EM, granular bile (Byler’s bile) was seen suggesting PFIC type1 [Figure 2]. The child was on regular fat-soluble vitamin supplements, medium chain triglycerides, and ursodeoxycholic acid (UDCA) at a dose of 20 mg/kg/day. Ondansetron and rifampicin were also prescribed for the pruritus. However, the response was not satisfactory and hence partial internal biliary diversion through a cholecystojejunocolic anastomosis was done to relieve the pruritus by interrupting the enterohepatic circulation and decreasing the preload of the bile salts to the liver. A 15-cm loop of bowel was isolated from mid jejunum and this conduit was sutured in an isoperistaltic manner superiorly to the gall bladder and inferiorly to the anterior aspect of mid ascending colon. Full thickness of gallbladder was anastomosed to the serosa of the conduit with a single layer of 4.0 vicryl sutures. A single layer of serosa to serosa anastomosis was performed with 4.0 vicryl sutures between the conduit and the colon. The distal end of the jejunum was tapered prior to anastomizing to the colon to prevent colonic contents from entering the conduit [Figure 3]. Postoperatively, there was an unbelievable cessation of the pruritus. On follow-up after 2 years, he neither had pruritus nor jaundice and the skin changes including lichenification had disappeared [Figure 1b]. There was also an improvement in his height (97 cm) and weight (18 kg). He has intermittent diarrhea, which could probably be due to high concentration of bile salts in the intestine.

## DISCUSSION

PFIC 1 is a systemic inherited disorder with hepatic, intestinal, and pancreatic manifestations. The common clinical features of the PFIC group are jaundice in early infancy, hepatosplenomegaly, severe intractable pruritus, growth failure, and progression to cirrhosis. Diarrhea, epistaxis, pancreatitis, gallstones, and hearing loss are some additional features which may be seen in PFIC 1. The constant scratching and rubbing of the extremities resulting in marked thickening and lichenification of the skin on both hands and feet with enlargement of the fingers and toes, resembling those of manual laborers, has been reported in PFIC 1.[3]

This classical presentation was seen in our case [Figure 1]. Some characteristic biochemical features which help in identifying and differentiating PFIC1 from other familial cholestatic disorders include mild to moderate elevation of aminotransferases, low or normal GGT, normal serum alpha fetoprotein, low serum cholesterol, and elevated serum and urine bile acids. The pathological feature of bland cholestasis and cirrhosis with granular bile on EM is also a manifestation of PFIC 1.[4] The management includes nutritional support using medium chain triglycerides, water- and fat-soluble vitamins, and calcium supplementation. The distressing problem in PFIC is the intractable pruritus that may not respond to therapy as in our patient. UDCA is recommended in a dose of 20 mg/kg/day. Ondansetron, rifampicin, phenobarbitone, naloxone, and propofol have all been tried with variable results. Surgical options such as biliary diversion have shown some beneficial effects in PFIC. They decrease the amount of bile acids in the enterohepatic circulation by 50% and thereby decrease preload to the biliary canaliculus.[5] The best results such as relief of pruritus, increase in growth velocity, and slowing or arrest of disease progression are observed when surgery is done early in the course of the disease before severe fibrosis. In 1988, Whitington and Whitington performed cholecystojejunocutaneostomy as a form of partial external biliary diversion for relieving pruritus by increasing the elimination of accumulated bile acids.[6] Ileal bypass procedure was proposed to combat the problem of stoma.[7] Bustorff-Silva *et al*.[2] reported and performed cholecystojejunocolonic anastomosis in two children as a partial internal biliary diversion to avoid the stigma and complications of stoma and also to prevent malabsorption. Biliary diversion may also delay the need for liver transplant. To the best of our knowledge, our patient who underwent cholecystojejunocolic anastomosis and partial biliary diversion in view of intractable pruritis showing a good response is the third case to be reported for the procedure. The clinical and laboratory parameters in this child following biliary diversion were so gratifying, making one consider biliary diversion as the treatment for children with PFIC 1. However, more studies and long-term follow-up is necessary before universal recommendation.
